# Single low‐dose decitabine as frontline therapy of acute myeloid leukaemia, with venetoclax salvage

**DOI:** 10.1111/jcmm.18592

**Published:** 2024-10-22

**Authors:** Ciprian Jitaru, Mareike Cathrina Peters, Lovisha Aggarwal, Anamaria Bancos, Adrian Bogdan Tigu, Diana Cenariu, Cristina Selicean, Sergiu Pasca, Vlad Moisoiu, Petra Rotariu, Maria Santa, Sabina Iluta, Rares Drula, David Kegyes, Aranka Kurtus, Mihnea Zdrenghea, Lukasz Gondek, Ciprian Tomuleasa, Gabriel Ghiaur

**Affiliations:** ^1^ Research Center for Advanced Medicine MedFUTRE Iuliu Hatieganu University of Medicine and Pharmacy Cluj‐Napoca Romania; ^2^ Department of Hematology The Oncology Institute ‘Prof. Dr. Ion Chiricuta’ Cluj‐Napoca Romania; ^3^ Sidney Kimmel Comprehensive Cancer Center Johns Hopkins University School of Medicine Baltimore Maryland USA; ^4^ Academy of Romanian Scientists Bucharest Romania; ^5^ Department of Hematology Municipal Hospital Odorheiu Secuiesc Odorheiu Secuiesc Romania

## 1 INTRODUCTION

The introduction of the combination therapy hypomethylating agents (HMA) with venetoclax established a new standard of care for patients with de novo AML who are unfit for intensive cytotoxic treatment.^1^ Standard dose HMA (pulse‐cycled administration for 5–7 days every 4 weeks) used as a single agent or in combination exerts indiscriminate cytotoxic effects on both tumour cells and healthy haematopoietic tissue^2^, ^3^ Historically, the dosing schedule of HMA treatments was initially established based on the maximum tolerated dose (decitabine [DEC]: 1500–2500 mg/m^2^), proving impractical in AML patients due to prolonged myelosuppression.^4^ Empiric down titration yielded clinically effective doses of HMAs with acceptable side effects (DEC: 20 mg/kg/day).^5^, ^6^ As an epigenetic modulator at non‐cytotoxic doses (0.1–0.2 mg/kg/day), DEC incorporates into newly synthesized DNA and depletes the chromatin‐modifying enzyme DNA methyltransferase 1 (DNMT1). Hypomethylation of tumour cell‐specific dysregulated DNA methylation patterns leads to changes in gene expression, restoring cell differentiation in favour of cell proliferation.^7^ Simultaneously, normal haematopoietic stem cells are stimulated to self‐renew,^8^ while committed progenitors are prompted to differentiate, thus limiting toxic effects on healthy haematopoietic cells.^8^ Higher frequency administrations of HMAs at lower concentrations might decrease treatment‐related complications, thereby providing a reasonable treatment strategy for extremely unfit patients with AML.

## 2 METHODS

For in vitro studies, seven AML cell lines were used (MV4‐11, TF‐1, THP1, MOLM‐14, OCI‐AML3, OCI‐AML5 and UCSD‐AML1). Cells were cultured in RPMI Medium 1640 (Gibco) supplemented with 10% heat‐inactivated fetal bovine serum, 2 mM L‐glutamine (Gibco) and 100 units/mL Pen/Strep (Gibco) at 37°C, 5% CO2. Cell viability post‐drug treatment was evaluated using the CellTiter 96 AQueous One Solution kit (Promega). Cells were seeded in 96‐well plates and treated with 0.5 μM DEC and venetoclax (VEN) starting from 10 μM up to eight 10‐fold dilutions, or DEC alone, using seven ten‐fold dilutions starting from 5 μM. In all drug experiments, corresponding cell‐free reactions were established for background correction. Triplicate measurements were conducted for all dose–response experiments. The absorbance values were read using CLARIOstar Plus Microplate Reader. Data were analysed using MS Excel and visualized using GraphPad Prism. The combination index was calculated using the Bliss independence formula to assess drug synergism.^13^

Patients were deemed ‘unfit for intensive therapy’ clinically by the treating physician. The study protocol was approved by the ethics committee of the Oncology Institute ‘Prof. Dr. Ion Chiricuţă’ Cluj‐Napoca, Romania.

## 3 RESULTS

**FIGURE 1:**
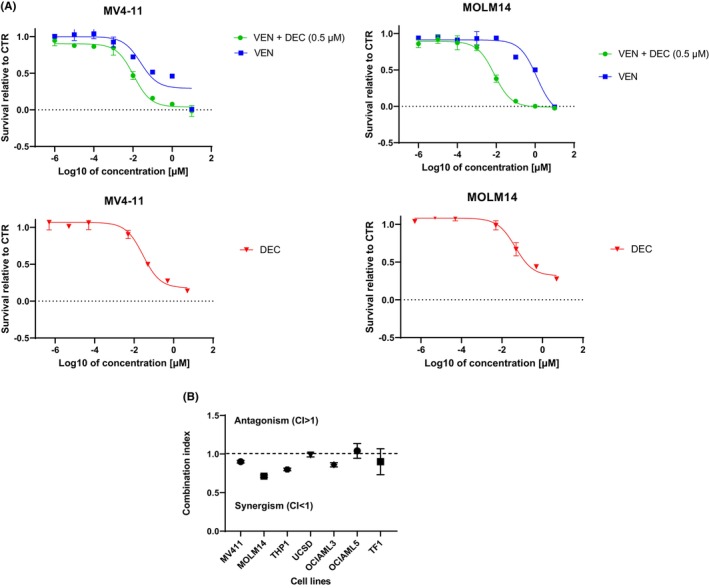
(A) Dose–response curves of AML cell lines MOLM14 and MV4‐11 to VEN in the presence and absence of decitabine (DEC), as well as to decitabine only (range: 5 μM–0.005 nM), at 24 h. (B) Combination Index of AML cell lines treated with VEN + LDDec.^13^

It was previously shown that AML cells but not normal haematopoietic cells are sensitive to low‐dose DEC (0.5 μM). Since current treatment protocols use a combination of HMA and VEN, we tested if LDDec sensitizes AML cells to VEN. On two AML cells lines, MV4‐11 and MOLM14 treatment with 0.5 μM DEC improves VEN sensitivity (Figure 1A). On multiple AML cell lines we show that 0.5 μM DEC and VEN are synergistic (Figure 1B). Since the two drugs act synergistically in vitro, we tested the feasibility of the combination in patients with AML.

**FIGURE 2:**
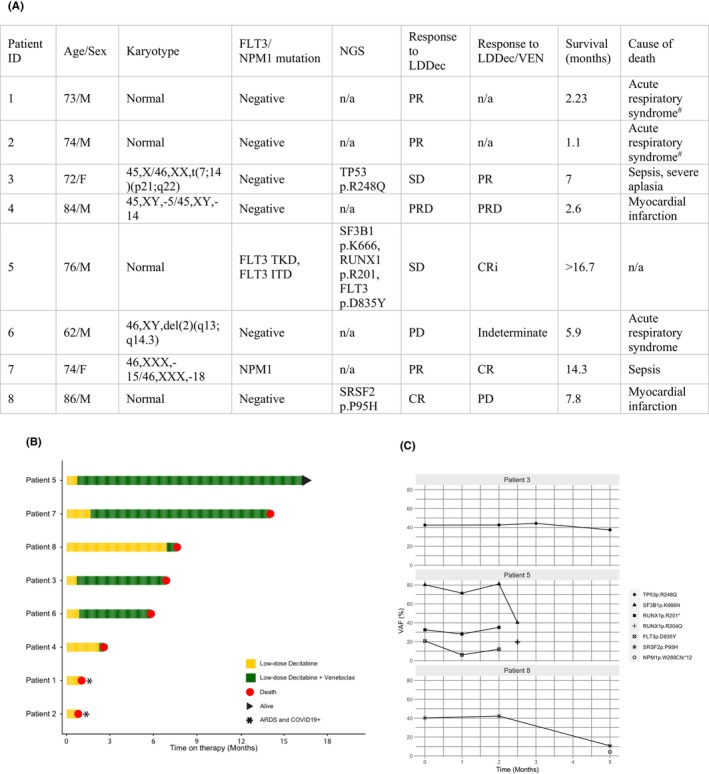
(A) Patient characteristics, (B) clinical evolution and (C) clonal evolution with LDDec+/− VEN treatment.

For clinical studies, eight elderly patients (median age: 74 years) with newly diagnosed AML ineligible for standard chemotherapy were treated with LDDec regimen, between July 2021 and May 2023. Treatment‐naive patients received LDDec (0.2 mg/kg) by intravenous administration, twice weekly on non‐consecutive days, continuously until disease progression. In addition to LDDec, six patients received daily VEN (p.o., standard dose: 400 mg/day, following ramp‐up) as salvage therapy for relapsed/refractory disease. The median overall survival was 6.5 months (194.5 days), while two patients exceeded 1 year of survival. Single‐agent LDDec resulted in induction of complete remission in one patient (12.5%), and partial remission in three patients (37.5%). The remaining four patients experienced stable disease (*N* = 2; 25%), resistant disease (*N* = 1; 12.5%) or progressive disease (*N* = 1; 12.5%). Venetoclax salvage therapy led to complete remission/complete remission with incomplete hematologic recovery in two patients (33%) and partial remission in one patient (16.7%) (Figure 2B). The median duration of treatment with single agent LDDec was 4.5 weeks, and with combination with venetoclax 24.2 weeks.

Complex karyotype and chromosomal monosomies, translocations and deletions were the most common cytogenetic abnormalities at diagnosis (Figure 2A). Next‐generation sequencing (NGS) was performed on only three patients as this procedure is not standard of care at our institution yet. NGS study of patient 3 showed the presence of a *TP53 p.R248Q* clone (VAF = 42.47%). The clone size remained relatively stable during treatment with either LDDec or LDDec/VEN (VAF = 42.50% at 2 months, VAF = 44.29% at 3, and VAF = 37.38% at 5 months), despite induction of partial remission under LDDec/VEN treatment. Patient 8, at diagnosis, presented with a *SRSF2 p.P95H* mutation (VAF = 40.21%). LDDec proved effective in reducing the size of the clone, reaching VAF = 10.68% at 5 months, paralleled by complete remission with incomplete hematologic recovery after 2 weeks of treatment. The combination of LDDec/VEN led to the reduction of an *SF3B1 p.K6666N* clone (VAF = 80.26%) after 10 weeks of treatment (40.24%) of patient 5 (Figure 2C). Simultaneously, bone marrow blasts were absent in this patient.

## 4 DISCUSSION

The survival benefit and safety of LDDec therapy for patients with myeloid malignancies were evaluated through a small series of clinical trials in recent years.^5^, ^9^, ^10^, ^11^ Low‐dose DEC as an alternative for elderly patients with AML, either as first or second‐line therapy, addressing a range of myeloid disorders (primary AML, secondary AML, CMML, MDS and MPN) showed complete remission/hematologic improvement in 43% of all patients, and 40% of AML patients.^9^ Recently, Levitz et al. reported that 63% of MDS/AML patients treated with LDDec/VEN, compared to 67% of patients receiving standard HMA/VEN achieved complete remission/complete remission with incomplete hematologic recovery.

In this case series, 50% of patients treated with single‐agent LDDec achieved complete or partial remission. Considering the synergistic effect of venetoclax with LDDec in vitro, we decided to add VEN to LDDec in patients unable to achieve or maintain remission with LDDec alone. The addition of venetoclax as salvage was associated with an overall response rate of 50%. Overall survival of all treated patients was 6.5 months. To place these data in perspective, patients with newly diagnosed AML treated at the same hospital had a median survival of 3.4 months with best supportive care (mean age: 80.6 years), thus inferior to LDDec +/− VEN.^12^ A comparison between LDDec +/− VEN and standard HMA regimens is impossible at present given the different clinical characteristics of these patients and the difference in route of administration of HMA (subcutaneous in standard of care HMA and intravenous for LDDec regimens). The low number of patients presented here is an important limitation. Thus, further studies using LDDec +/− VEN should employ larger patient cohorts, ideally in a randomized design comparing standard doses of DEC. Short of such a trial design, the data presented here adds to the growing body of evidence supporting LDDec as a reasonable alternative to standard doses. In addition, correlative studies using serial NGS would allow in‐depth analysis of the evolution of clonal architecture.

## 5 CONCLUSION

This case series provides insights into the feasibility of treating elderly patients with newly diagnosed AML with first‐line LDDec, with VEN salvage therapy, while highlighting the need for ongoing clinical studies to determine the most beneficial use of noncytotoxic therapy.

## AUTHOR CONTRIBUTIONS

**Ciprian Jitaru:** Data curation (lead); formal analysis (lead); investigation (lead); writing – original draft (lead); writing – review and editing (equal). **Mareike Cathrina Peters:** Formal analysis (lead); investigation (equal); writing – original draft (lead); writing – review and editing (lead). **Lovisha Aggarwal:** Data curation (equal); investigation (equal); writing – review and editing (equal). **Anamaria Bancos:** Data curation (equal); investigation (equal); writing – review and editing (equal). **Adrian‐Bogdan Tigu:** Data curation (equal); investigation (equal); writing – review and editing (equal). **Diana Cenariu:** Data curation (equal); investigation (equal); writing – review and editing (equal). **Cristina Selicean:** Data curation (equal); investigation (equal); writing – review and editing (equal). **Sergiu Pasca:** Investigation (equal); writing – review and editing (equal). **Vlad Moisoiu:** Visualization (lead); writing – review and editing (equal). **Petra Rotariu:** Data curation (equal); investigation (equal). **Maria Santa:** Data curation (equal); investigation (equal). **Sabina Iluta:** Data curation (equal); investigation (equal); writing – review and editing (equal). **Rares Drula:** Data curation (equal); investigation (equal). **David Kegyes:** Investigation (equal). **Aranka Kurtus:** Data curation (equal); investigation (equal). **Mihnea Zdrenghea:** Project administration (equal); supervision (equal). **Lukasz Gondek:** Supervision (equal); writing – review and editing (equal). **Ciprian Tomuleasa:** Conceptualization (equal); data curation (equal); investigation (equal); project administration (equal); supervision (equal); validation (equal); writing – review and editing (equal). **Gabriel Ghiaur:** Conceptualization (lead); project administration (lead); supervision (lead); validation (equal); writing – review and editing (lead).

## FUNDING INFORMATION

DK, CT, BT, and RD are supported by a national grant of the Romanian Academy of Scientists (Academia Oamenilor de Stiinta din Romania) 2023–2024. MCP is funded by a national grant of the Romanian Society for Bone Marrow Transplantation (Nr. 2/01.03.2022) and an internal grant of the Iuliu Hatieganu University of Medicine and Pharmacy—School of Medicine. DK is funded, in part, by a grant from Iuliu Hatieganu University—School of Medicine, as well as by two grants awarded by the Romanian National Ministry of Research, Innovation, and Digitalization: Project 539PED (PN‐III‐P2‐2.1‐PED‐2019‐3640), Project PD 122 (PN‐III‐P1‐1.1.‐PD‐2019‐0805), as well as by an international collaborative grant of the European Economic Space between Romania and Iceland 2021–2023: ‘Cooperation strategy for knowledge transfer, internationalization and curricula innovation in the field of research education at the 3rd level of study—AURORA.’ AB is funded by an internal grant of the Iuliu Hatieganu University of Medicine and Pharmacy—School for Doctoral Studies. GG is supported by a grant awarded by the Romanian National Ministry of Research, Innovation, and Digitalization: Project PN‐III‐P4‐ID‐PCE‐2020‐1118. CT is also supported by three grants awarded by the Romanian National Ministry of Research, Innovation, and Digitalization; Project No. PN‐III‐P4‐ID‐PCCF‐2016‐0112 within PNCDI III, Project No. PN‐III‐P1‐1.1‐TE‐2019‐0271 for Young Research Teams 2020–2022 and Project No. 13‐BM/2020 (PN‐III‐CEI‐BIM‐PBE‐2020‐0016) within PNCDI I.

## CONFLICT OF INTEREST STATEMENT

The authors declare that they have no known competing financial interests or personal relationships that could have appeared to influence the work reported in this paper.

## Data Availability

The data that support the findings of this study are available from the corresponding author upon reasonable request.
